# Investigation of pathogen distribution, clinical characteristics, and risk factors of neonatal urinary tract infections

**DOI:** 10.3389/fped.2025.1672899

**Published:** 2025-11-19

**Authors:** Bo Jiang, Xiuyi Gao

**Affiliations:** 1 Department of Urology, Affiliated Hospital of Beihua University, Jilin, Jilin, China; 2 Department of Neonatology, Jilin Central Hospital, Jilin, Jilin, China

**Keywords:** neonate, urinary tract infection, pathogen, risk factor, trend analysis

## Abstract

**Objective:**

To investigate the annual variation in pathogen distribution, clinical characteristics, and associated risk factors of neonatal urinary tract infections (UTIs), providing a scientific basis for clinical diagnosis and treatment.

**Methods:**

A retrospective analysis was conducted on clinical data from 134 neonatal UTI cases admitted to our hospital between January 2021 and December 2024. The distribution of pathogens, bacterial resistance patterns, clinical manifestations, and laboratory findings were compared in different years. Multivariable logistic regression was employed to identify risk factors associated with neonatal UTIs.

**Results:**

(1) Among the 134 pediatric patients, 86 were male and 48 were female; 52 (38.8%) were preterm, while 82 (61.2%) were full-term. The incidence rates of UTIs from 2021 to 2024 were 8.2%, 9.1%, 10.3%, and 11.2%, respectively, with a statistically significant difference across the years (*P* < 0.05). (2) The primary clinical manifestations included fever (72 cases, 53.7%), crying (65 cases, 48.5%), and poor feeding (58 cases, 43.3%). (3) From 2021 to 2024, the detection rate of Gram-negative bacteria exhibited a significant downward trend (75.8%, 70.3%, 65.1%, and 61.2%, *P* < 0.05), with *Escherichia coli* showing a yearly decline (*P* < 0.05). Conversely, the detection rate of Gram-positive bacteria demonstrated a significant upward trend (18.2%, 24.3%, 28.6%, and 32.7%, *P* < 0.05), with coagulase-negative staphylococci increasing annually (*P* < 0.05). (4) The detection rate of Gram-positive bacteria was significantly higher in the low birth weight group than in the normal birth weight group (*P* < 0.05). (5) The antibiotic resistance rates of *E. coli* and *Enterococcus faecium* showed a progressive increase over the study period (*P* < 0.05). (6) Multivariable analysis identified low birth weight (OR = 2.831, 95% CI: 1.562–5.121), indwelling urinary catheter (OR = 3.452, 95% CI: 1.891–6.282), and maternal infection during pregnancy (OR = 2.154, 95% CI: 1.233–3.762) as independent risk factors for neonatal UTIs.

**Conclusion:**

The incidence of neonatal UTIs shows a progressive upward trend in recent years, accompanied by a significant shift in pathogen spectra. Particular attention should be given to high-risk factors such as low birth weight and indwelling urinary catheters, necessitating targeted preventive measures.

## Introduction

Neonatal urinary tract infection (UTI), a prevalent infectious disease in the neonatal period, has exhibited significant changes in incidence rates and clinical characteristics in recent years (1). Global data indicate that the incidence of neonatal UTIs ranges from 0.1% to 1% in developed countries, whereas it can reach 2%−4% in developing countries, with a persistent upward trend (2). Studies showed that the detection rate of neonatal UTIs among febrile neonates increased from 7% in 2015 to 11% in 2022, underscoring the need for heightened clinical vigilance (3, 4).

The distribution of pathogens in neonatal UTIs exhibits significant regional and temporal variations (5). Studies from Europe and North America showed that the proportion of Gram-positive bacteria in neonatal UTIs increased from 15% in 2010 to 35% in 2022, with coagulase-negative staphylococci and *Enterococcus faecalis* being the predominant species (6). In contrast, data from the Asia-Pacific region revealed that while Gram-negative bacteria remained dominant, their proportion declined from 80% in 2015 to 65% in 2022, accompanied by an increasingly diverse distribution of strains (7). This dynamic shift in pathogen spectrum not only complicates empirical treatment but also poses new challenges for the development of clinical therapeutic strategies.

Global multicenter studies have revealed a nearly 40% increase in the detection rate of ESBL-producing *Escherichia coli* over the past five years, while the detection rate of carbapenem-resistant *Klebsiella pneumoniae* tripled compared to a decade ago (8, 9). Additionally, research indicates that the prevalence of methicillin-resistant *Staphylococcus aureus* (MRSA) in neonatal UTIs has reached 20%–30% and continues to rise (10, 11). Recent advances in the study of neonatal UTI risk factors (12) have identified not only anatomical and physiological contributors but also the potential roles of maternal-infant microbiome imbalance, perinatal antibiotic exposure, and medical device-related factors in increasing the risk of neonatal UTIs (13).

The aim of this study was to conduct a systematic analysis of neonatal UTI cases over four years at our hospital, with a particular focus on the annual trends in pathogen distribution and their correlation with clinical manifestations. Additionally, a multivariable analysis was employed to explore relevant risk factors in depth. This holds significant implications for improving the prevention and treatment strategies of neonatal UTIs. Compared with previous studies, the novelty of this research lies in its first-time application of annual sequence analysis to systematically evaluate shifts in pathogen distribution, its multidimensional approach to uncovering the correlation between clinical manifestations and pathogen distribution, and the establishment of a predictive model for risk factors.

## Materials and methods

### Study design and patient screening

This retrospective study was approved by the ethics committee of Jilin Central Hospital and complied with the requirements of the Declaration of Helsinki. The study period spanned from January 2021 to December 2024, during which clinical data were collected on neonates diagnosed with UTIs at our hospital. A preliminary cohort of 159 cases was identified and subsequently screened based on the following inclusion and exclusion criteria:

Inclusion criteria: (1) Neonates aged 0–28 days after birth; (2) Diagnosed with UTIs according to the criteria outlined in the 5th edition of Practice of Neonatology (UTI diagnosis required both clinical manifestations and laboratory criteria, specifically including clean midstream urine culture ≥10^5^ CFU/mL or catheterized specimen ≥5  ×  10^4^ CFU/mL; the child exhibited at least one symptom such as fever, crying, or poor feeding, and catheter-associated UTI was defined as UTI occurring 48 h after indwelling catheter) and presented with corresponding clinical symptoms (14); (3) Hospitalized for ≥72 h; (4) Complete clinical data available, including medical history, laboratory tests, and imaging findings; (5) Male infants who had not undergone circumcision.

Exclusion criteria: (1) Administration of antibiotics for >24 h prior to admission; (2) Concurrent severe infections at other sites; (3) Congenital malformations of the urinary system; (4) Severe congenital heart disease; (5) Immunodeficiency or metabolic disorders; (6) Incomplete clinical data; (7) Cases of automated discharge or transfer to another hospital.

**Figure 1:**
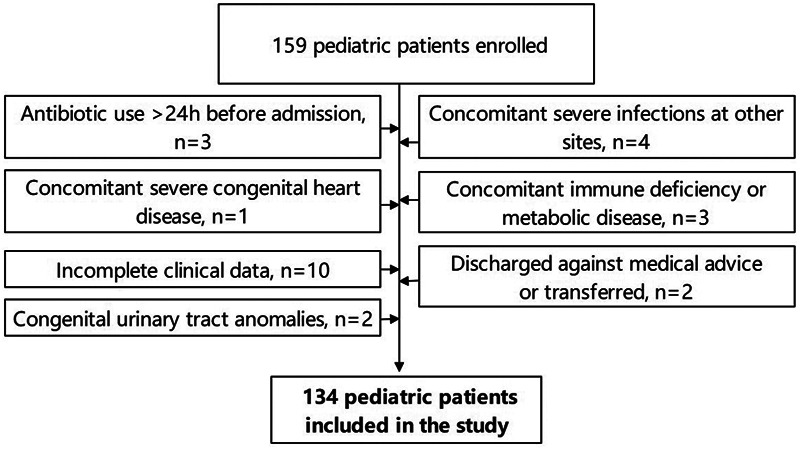
Flow chart of patient selection.

Following the screening of these criteria, a total of 134 neonates were included in the study (Figure 1), including 92 from the neonatal ward and 42 from the neonatal intensive care unit (NICU). Among them, 89 cases (66.4%) of urine cultures were collected by catheterization and 45 (33.6%) by clean-catch midstream urine collection. No suprapubic aspiration cases were included. Specimens were immediately sent for testing and inoculation culture was completed within 2 h.

Control group selection: To further analyze risk factors, hospitalized neonates during the same period were selected as the control group. Specific inclusion criteria were: (1) Neonates hospitalized in the Department of Neonatology during the same period (January 2021 to December 2024); (2) No occurrence of UTI during hospitalization; (3) Complete clinical data; (4) Matched 1:1 with the UTI group based on sex, gestational age (±1 week), and year of admission. In total, 134 neonates were ultimately included in the control group, including 84 males and 50 females, of whom 32 were preterm and 102 were term infants. The annual distribution was 30 cases in 2021, 32 cases in 2022, 35 cases in 2023, and 37 cases in 2024.

### Data collection

Relying on the hospital's HIT system, pediatric patients were categorized into groups according to the year: 2021 (*n* = 30), 2022 (*n* = 32), 2023 (*n* = 35), and 2024 (*n* = 37). Subsequently, the following data were collected for each patient: (1) General information, including sex and gestational age; (2) Clinical characteristics, with an annual statistical analysis of varying clinical manifestations of neonatal UTIs, such as fever, crying, poor feeding, and vomiting; (3) Annual trends in pathogen distribution: the detection rates of Gram-positive bacteria, Gram-negative bacteria, and fungi were analyzed across different years, followed by the assessment of the distribution of predominant pathogenic species; (4) Evolution of antimicrobial resistance patterns of major pathogens [resistance classification refers to the following standards: multiple-drug resistance (MDR) denotes resistance to ≥3 categories of antimicrobial drugs, extensively drug resistance (XDR) denotes susceptibility to only 1–2 categories of drugs, and pan drug resistance (PDR) denotes resistance to all tested drugs]; (5) Identification of risk factors for neonatal UTIs through logistic regression analysis.

### Outcome measures and statistical analysis

This study primarily compared variations in the clinical manifestations of neonatal UTIs across different years, differences in the distribution of pathogens over time, and temporal changes in the antibiotic resistance patterns of major pathogens. Additionally, potential risk factors associated with neonatal UTIs were analyzed using both univariate and multivariable methods.

All data collection was conducted using Excel 2021, while data processing was performed with SPSS 22.0. Categorical data were expressed as percentages, with intergroup differences assessed using the chi-square test. Continuous data were tested for normal distribution and presented as mean ± standard deviation, with intergroup comparisons analyzed using the independent sample *t*-test. Risk factor analysis was conducted using multivariable logistic regression. A *P*-value < 0.05 was considered statistically significant.

## Results

### Analysis of general information

Among the 134 pediatric patients, 86 were male and 48 were female; 52 (38.8%) were preterm, while 82 (61.2%) were full-term. The age range was 1–28 days, with a mean age of (12.6 ± 5.6) days.

### Analysis of annual changes in the clinical characteristics of neonatal UTIs in different years

**Figure 2:**
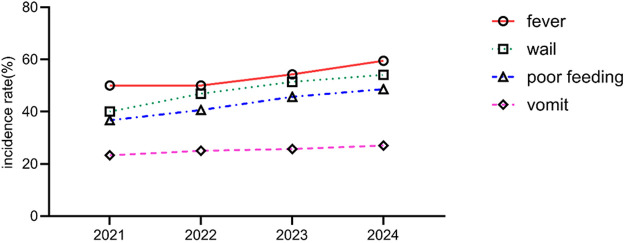
Analysis of annual changes in the clinical characteristics of neonatal UTIs in different years.

During the study period, 1,458 newborns were admitted, including 134 cases of UTI, with an overall incidence rate of 9.2%. A total of 116 cases (86.6%) underwent urinary tract ultrasound examination, revealing congenital anomalies of the kidney and urinary tract (CAKUT) in 12 cases (10.3%). The incidence of non-*Escherichia coli* infections was significantly higher in pediatric patients with CAKUT compared to those without anomalies (*P* = 0.041). Based on onset timing, 96 cases (71.6%) were community-acquired infections (diagnosed within 48 h of admission), while 38 cases (28.4%) were hospital-acquired infections (onset after 48 h of admission). Among hospital-acquired infections, catheter-associated UTIs (occurring after indwelling catheterization >48 h) accounted for 22 cases (57.9%). The proportion of Gram-positive bacteria was higher in the hospital-acquired infection group than in the community-acquired infection group (*P* = 0.028) (Table 1). Regarding the total incidence, from 2021 to 2024, the overall number of neonatal UTI cases exhibited an increasing trend. Clinical manifestations such as fever, crying, poor feeding, vomiting, and abdominal distension in the included infants showed a year-on-year rise. A comparison of the data revealed statistically significant differences in fever, crying, and poor feeding (*P* < 0.05), as shown in Table 2 and Figure 2.

**Table 1:** Annual trend analysis of CAKUT, hospital-acquired infections, and catheter-associated UTI.

Subgroup type	2021 (*n* = 30)	2022 (*n* = 32)	2023 (*n* = 35)	2024 (*n* = 37)	*χ* ^2^	*P*
CAKUT detection	2 (6.7)	3 (9.4)	3 (8.6)	4 (10.8)	1.245	0.642
Hospital-acquired Infection	7 (23.3)	9 (28.1)	10 (28.6)	12 (32.4)	2.156	0.458
Catheter-associated UTI	4 (13.3)	5 (15.6)	6 (17.1)	7 (18.9)	1.876	0.512

**Table 2:** Analysis of annual changes in the clinical characteristics of neonatal UTIs in different years [*n* (%)].

Clinical manifestations	2021 (*n* = 30)	2022 (*n* = 32)	2023 (*n* = 35)	2024 (*n* = 37)	χ^2^	*P*
Fever	15 (50.0)	16 (50.0)	19 (54.3)	22 (59.5)	4.562	0.035
Crying	12 (40.0)	15 (46.9)	18 (51.4)	20 (54.1)	5.124	0.028
Poor feeding	11 (36.7)	13 (40.6)	16 (45.7)	18 (48.6)	4.876	0.031
Vomiting	8 (26.7)	9 (28.1)	11 (31.4)	12 (32.4)	3.245	0.086
Abdominal distension	7 (23.3)	8 (25.0)	9 (25.7)	10 (27.0)	2.987	0.092

### Annual trend analysis of pathogen distribution in neonatal UTIs

**Figure 3:**
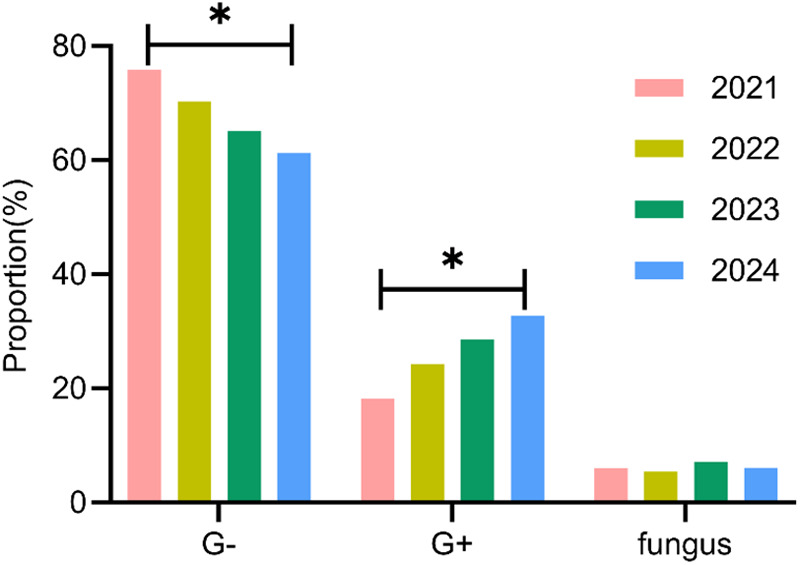
Annual trend analysis of pathogen distribution in neonatal UTIs. *P < 0.05.

A total of 161 pathogens were isolated from 134 pediatric patients, including 115 cases (85.8%) of single infection and 19 cases (14.2%) of mixed infection. In 2021, 30 neonatal UTI cases identified 33 distinct pathogens; in 2022, 32 cases revealed 37 pathogens; in 2023, 35 cases detected 42 pathogens; and in 2024, 37 cases identified 49 pathogens. An analysis of the temporal shifts in the prevalence of different pathogen types showed a significant decrease in the proportion of Gram-negative bacteria over the years (*P* < 0.05), whereas the proportion of Gram-positive bacteria demonstrated a significant increase (*P* < 0.05). No significant difference was observed in the fungal pathogen proportion (*P* > 0.05) (Table 3, Figure 3).

**Table 3:** Annual trend analysis of pathogen distribution in neonatal UTIs [*n* (%)].

Pathogens	2021 (*n* = 33)	2022 (*n* = 37)	2023 (*n* = 42)	2024 (*n* = 49)	χ^2^	*P*
Gram-negative bacteria	25 (75.8)	26 (70.3)	27 (65.1)	30 (61.2)	8.624	0.012
Gram-positive bacteria	6 (18.2)	9 (24.3)	12 (28.6)	16 (32.7)	7.856	0.015
Fungi	2 (6.0)	2 (5.4)	3 (7.1)	3 (6.1)	1.245	0.458

### Analysis of the temporal distribution changes of major pathogens of different types and years

**Figure 4:**
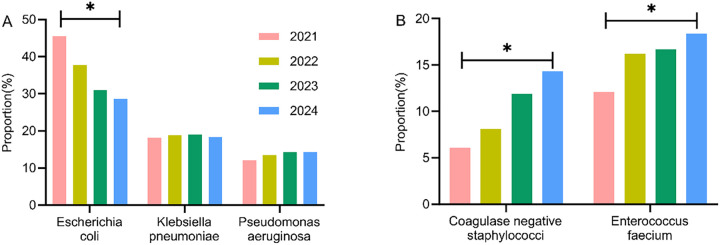
Analysis of the temporal distribution changes of major pathogens of different types and years. **(A)** Gram-negative bacteria; **(B)** Gram-positive bacteria.

The major pathogens of Gram-negative and Gram-positive bacteria were extracted, and their distribution changes over different years were analyzed. The results indicated a decreasing trend in *E. coli* among Gram-negative bacteria, with statistically significant differences observed between years (*P* < 0.05). Conversely, coagulase-negative staphylococci and *Enterococcus faecium* in Gram-positive bacteria exhibited an increasing trend, with statistically significant differences (*P* < 0.05), as shown in Table 4 and Figures 4A, B.

**Table 4:** Analysis of the temporal distribution changes of major pathogens of different types and years [*n* (%)].

Pathogen type	2021 (*n* = 33)	2022 (*n* = 37)	2023 (*n* = 42)	2024 (*n* = 49)	χ^2^	*P*
Gram-negative bacteria						
*Escherichia coli*	15 (45.5)	14 (37.8)	13 (31.0)	14 (28.6)	6.847	0.021
*Klebsiella pneumoniae*	6 (18.2)	7 (18.9)	8 (19.0)	9 (18.4)	1.234	0.452
*Pseudomonas aeruginosa*	4 (12.1)	5 (13.5)	6 (14.3)	7 (14.3)	1.156	0.468
Gram-positive bacteria						
Coagulase-negative staphylococci	2 (6.1)	3 (8.1)	5 (11.9)	7 (14.3)	5.678	0.025
*Enterococcus faecium*	4 (12.1)	6 (16.2)	7 (16.7)	9 (18.4)	4.987	0.032

### Analysis of the temporal trends in the antibiotic resistance rates of major pathogens

**Figure 5:**
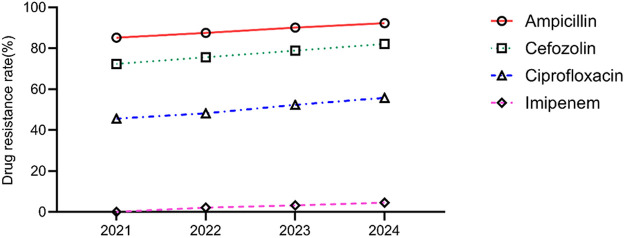
Analysis of the trends in the antibiotic resistance rates of *Escherichia coli.*

**Figure 6:**
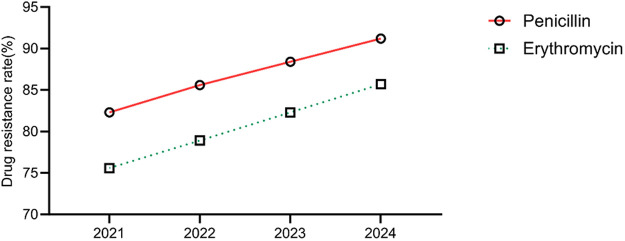
Analysis of the trends in the antibiotic resistance rates of *Enterococcus faecium.*

The annual trends in the antibiotic resistance rates of *E. coli* and *E. faecium* were analyzed. The results indicated a significant increase in the resistance rates of *E. coli* to ampicillin, cefazolin, ciprofloxacin, and imipenem over the years (*P* < 0.05). Similarly, *E. faecium* exhibited a consistent rise in resistance to penicillin and erythromycin, with statistically significant differences (*P* < 0.05) (Tables 5, 6, Figures 5, 6).

**Table 5:** Analysis of the trends in the antibiotic resistance rates of Escherichia coli [*n* (%)].

Antibiotics	2021 (*n* = 33)	2022 (*n* = 37)	2023 (*n* = 42)	2024 (*n* = 49)	χ^2^	*P*
Ampicillin	85.2	87.5	90.1	92.3	5.234	0.028
Cefazolin	72.4	75.6	78.9	82.1	4.987	0.031
Ciprofloxacin	45.6	48.2	52.4	55.8	4.658	0.035
Imipenem	0.0	2.1	3.2	4.5	3.987	0.042
Gentamicin	28.6	30.2	31.5	32.4	1.236	0.087
Amikacin	12.3	13.1	13.8	14.5	1.015	0.124

**Table 6:** Analysis of the trends in the antibiotic resistance rates of Enterococcus faecium [*n* (%)].

Antibiotics	2021 (*n* = 33)	2022 (*n* = 37)	2023 (*n* = 42)	2024 (*n* = 49)	χ^2^	*P*
Penicillin	82.3	85.6	88.4	91.2	5.123	0.029
Erythromycin	75.6	78.9	82.3	85.7	4.856	0.033
Vancomycin	0.0	0.0	0.0	0.0	–	–
Teicoplanin	0.0	0.0	0.0	0.0	–	–

### Analysis of pathogen distribution and antibiotic resistance differences among subgroups with different birth weights

Newborns were categorized into two groups based on birth weight: low birth weight group (<2,500 g, *n* = 52) and normal birth weight group (≥2,500 g, *n* = 82). The distribution of pathogens and antibiotic resistance differences were analyzed between the two groups. The results indicated that the detection rate of Gram-positive bacteria in the low birth weight group was significantly higher than that in the normal birth weight group (*P* < 0.05), with notable differences in the proportions of coagulase-negative staphylococci and *E. faecium* (*P* < 0.05) (Table 7). Regarding antibiotic resistance, the low birth weight group exhibited a higher resistance rate of *E. coli* to cefazolin (89.2% vs. 76.5%) (*P* < 0.05), and a significant increase in the resistance rate of *E. faecium* to erythromycin (89.2% vs. 76.5%) (*P* < 0.05).

**Table 7:** Analysis of pathogen distribution and antibiotic resistance differences among subgroups with different birth weights.

Detection rate	Low birth weight group (*n* = 52)	Normal birth weight group (*n* = 82)	χ^2^	*P*
Detection rate of Gram-positive bacteria	38.5	24.4	5.232	0.014
Coagulase-negative staphylococci	18.2	8.5	4.226	0.021
*Enterococcus faecium*	15.4	7.3	3.269	0.036

### Analysis of risk factors for neonatal UTIs

Potential risk factors for neonatal UTIs, including birth weight, preterm birth, cesarean section, and maternal infections during pregnancy, were included. A univariate analysis was conducted, with the occurrence of neonatal UTIs as the dependent variable and the aforementioned factors as independent variables. Results revealed that low birth weight, preterm birth, cesarean section, maternal infections during pregnancy, and indwelling urinary catheters may be associated with neonatal UTIs (*P* < 0.05) (Table 8). Further multivariable logistic regression analysis identified that low birth weight, indwelling urinary catheters, and maternal infections during pregnancy were independent risk factors for neonatal UTI (*P* < 0.05) (Table 9).

**Table 8:** Univariate analysis of risk factors for neonatal UTI.

Risk factor	UTI group (*n* = 134)	Control group (*n* = 134)	χ^2^	*P*
Low birth weight	52 (38.8)	28 (20.9)	9.856	0.002
Preterm birth	48 (35.8)	32 (23.9)	8.624	0.003
Cesarean section	78 (58.2)	65 (48.5)	4.562	0.033
Maternal infections during pregnancy	45 (33.6)	25 (18.7)	7.856	0.005
Indwelling urinary catheters	38 (28.4)	15 (11.2)	12.458	<0.001

**Table 9:** Multivariable logistic regression analysis of risk factors for neonatal UTI.

Risk factor	B	*P*	OR	95% CI
Low birth weight	1.040	0.001	2.831	1.562–5.121
Indwelling urinary catheters	1.238	<0.001	3.452	1.891–6.282
Maternal infections during pregnancy	0.765	0.007	2.154	1.233–3.762

## Discussion

Neonatal UTI is one of the most common infectious diseases in the neonatal period (15). The incidence of neonatal UTI is about 0.1%−1%, with preterm infants demonstrating a higher prevalence of 3%–5%, and male neonates exhibit a higher incidence compared to females, primarily due to anatomical differences (16). Risk factors include congenital malformations of the urinary system, urinary catheter manipulation, immature immune function, and maternal infections (17). This study retrospectively analyzed the clinical characteristics, pathogen distribution, and evolution of antimicrobial resistance patterns of 134 neonatal UTI cases diagnosed between 2021 and 2024.

### Analysis of annual changes in the incidence and clinical manifestations of neonatal UTIs

This study identified fever (53.7%), crying (48.5%), and poor feeding (43.3%) as the main clinical manifestations of neonatal UTIs, aligning closely with the findings of Chen et al. (18), who developed a predictive model for neonatal UTIs by incorporating data from 2,235 pediatric patients and constructing a receiver operating characteristic (ROC) curve and highlighted fever and feeding-related symptoms as the most common clinical manifestations in pediatric patients with UTI. Furthermore, a comparative analysis of clinical characteristics across different years revealed an upward trend in these symptoms. This phenomenon may be associated with increasing bacterial resistance. Pathogens have progressively developed resistance to conventional antibiotics due to their widespread use, potentially exacerbating the severity of infection-related symptoms. Additionally, improved completeness of medical records and heightened clinical awareness among physicians may also contribute to this observed trend. However, this conclusion remains speculative; the precise mechanistic relationship between symptom severity and bacterial resistance, along with other potential influencing factors, warrants further in-depth investigation. Given these findings, we propose that fever, crying, and poor feeding, though relatively common in neonatal UTIs, lack specificity and are often mistaken for symptoms of other neonatal conditions, potentially leading to delayed diagnosis. Additionally, some pediatric patients in this study were diagnosed with CAKUT following UTI. Based on these findings, it is recommended that all neonates with a first episode of UTI undergo urinary tract ultrasound screening to reduce the rate of missed diagnoses.

This study further analyzed the annual trends of CAKUT, hospital-acquired infections, and catheter-associated UTIs. The results indicated that the incidence rates of these subgroups remained relatively stable throughout the study period (*P* > 0.05). Specifically, CAKUT detection rate ranged from 6.7% to 10.8%, hospital-acquired infections from 23.3% to 32.4%, and catheter-associated UTIs from 13.3% to 18.9%. These findings suggest that observed shifts in pathogen distribution and changes in antimicrobial resistance patterns were primarily driven by overall epidemiological trends rather than significant alterations in the proportions of specific high-risk subgroups.

### Distribution characteristics and antibiotic resistance trends of pathogens

This study primarily analyzed the temporal shifts in the distribution of pathogens among included pediatric patients. The findings indicated a declining trend in the overall detection rate of Gram-negative bacteria from 75.8% in 2021 to 61.2% in 2024, whereas the detection rate of Gram-positive bacteria exhibited an upward trend, rising from 18.2% to 32.7%. This phenomenon aligns with previous studies. Alcocer et al. (19) established diagnostic criteria for infections, revealed substantial age-related differences in pathogen distribution and antimicrobial resistance among pediatric patients, and highlighted significant annual variations in the pathogen distribution of UTIs, with a declining trend in the overall detection rate of Gram-negative bacteria, accompanied by an increasing prevalence of Gram-positive bacteria. The resistance rate to aminoglycosides (gentamicin and amikacin) remained stable and could be used as an option for empirical treatment. These findings suggest that shifts in pathogen distribution among neonatal UTI cases are a widespread global phenomenon (20), providing valuable insights for subsequent clinical diagnosis and treatment strategies. This study also found that pediatric patients with CAKUT were more susceptible to non-*E. coli* infections, suggesting that urinary tract structural abnormalities may alter the local microenvironment, thereby promoting the colonization of specific pathogens.

To further elucidate the temporal shifts in pathogen distribution, this study selected representative species, including *E. coli* and *E. faecium*, to analyze their annual variations. The results indicated a significant decline in the detection rate of *E. coli* (from 45.5% to 28.6%), whereas the detection rate of coagulase-negative staphylococci and *E. faecium* markedly increased. These findings align with multiple previous studies. Nakanishi et al. (21), through an analysis of pediatric patients with UTIs treated between April 2014 and March 2020, identified a shifting trend in the pathogen distribution of neonatal UTIs across different years, with a notably decreasing trend in *E. coli* infection rate. Belko et al. (22) have reported that neonates exhibit a higher incidence of UTIs during their first postnatal year, with some cases accompanied by intestinal and bladder dysfunction, and even urogenital anomalies, and that the etiologies of neonatal UTIs are diverse, among which alterations in antibiotic usage patterns may represent a significant contributing factor. We propose that the underlying factors driving this transformation are multifaceted. The empirical administration of broad-spectrum antibiotics targeting Gram-negative bacteria may suppress traditional dominant species such as *E. coli*, thereby creating ecological niches for opportunistic pathogens. Furthermore, advancements in diagnostic techniques have substantially enhanced the detection of atypical pathogens, which may also be a key contributor to the observed changes in pathogen spectra.

In addition, our findings indicated a concerning trend of antibiotic resistance of pathogens in neonates with UTIs, particularly the significantly increased resistance rate of *E. coli* to third-generation cephalosporins. Although the resistance rate to carbapenems remained relatively low in this study, it showed a gradual upward trend (from 0.0% to 4.5%), highlighting the worsening antimicrobial resistance landscape in neonatal UTIs. Similar findings were reported in other studies. González et al. (23) conducted a surveillance study on 492 neonates diagnosed with UTIs between January 2014 and November 2020, revealing a strong association between neonatal UTIs and underlying urinary tract anomalies, and they emphasized the critical importance of thorough diagnostic evaluation and screening in neonates with UTIs, with particular attention to antimicrobial resistance. This concern is especially pertinent in developing countries, where bacterial resistance in neonatal UTIs is more severe compared to developed countries, with carbapenem resistance rates in Asia and Africa reaching as high as 8.2%. We propose that the evolving antibiotic resistance patterns in neonatal UTIs stem from multiple factors. Irrational antibiotic use, compounded by the atypical presentation and rapid progression of neonatal infectious diseases, often prompts clinicians to favor empirical, broad-spectrum antibiotic therapy. This practice significantly accelerates the emergence and dissemination of resistant bacterial strains (24). Additionally, advancements in medical care have enabled the survival of more critically ill and complex cases, serving as a critical source for the emergence and dissemination of drug-resistant strains (25). Another possible reason may be the routine use of empirical treatment regimens for neonatal sepsis in this region, which typically combine ampicillin with aminoglycosides or third-generation cephalosporins. This antibiotic combination pattern may also contribute to the selective growth of drug-resistant strains.

### Analysis of risk factors

Finally, this study analyzed the risk factors for neonatal UTIs and identified low birth weight (OR = 2.831), indwelling urinary catheters (OR = 3.452), and maternal infections during pregnancy (OR = 2.154) as independent risk factors. These findings align with previous research. Yu et al. (26) revealed that among 258 children with suspected UTIs, hospitalization for ≥10 days, indwelling urinary catheters, a history of infection, congenital malformations, constipation, and extremely low birth weight (< 1,500 g) were independent risk factors for UTIs. Another study (27) demonstrated that the implementation of catheter bundle management significantly reduced the incidence of catheter-associated UTIs. We propose that neonates with low birth weight exhibit relatively underdeveloped immune functions and compromised skin and mucosal barrier integrity, increasing the risk of external bacterial infections, particularly in the presence of an indwelling urinary catheter. Additionally, maternal-infant transmission suggests that preventive strategies should be implemented earlier, emphasizing enhanced prenatal care, and recommending proactive intervention and treatment for pregnant women with infections. Based on these findings, the use of urinary catheters should be strictly limited to clinically indicated cases. For non-essential situations, alternative urine collection methods should be prioritized, and unnecessary catheters should be removed promptly. Although multivariable analysis did not identify preterm birth and cesarean section as independent risk factors for neonatal UTIs, existing research has indicated that these factors may indirectly elevate infection risk by disrupting neonatal immune function and microbiome balance. For instance, Bar et al. (28) conducted a retrospective analysis of medical records for infants with UTIs and noted that the prevalence of non-*E. coli* infections was significantly higher among cesarean-delivered infants (12 of 25 cases, 48%), revealing mode of delivery as the only variable associated with non-*E. coli* infections. Thus, close monitoring is recommended for these high-risk populations.

The incidence of neonatal UTIs shows a progressive upward trend in recent years, accompanied by a significant shift in pathogen spectra. Particular attention should be given to high-risk factors such as low birth weight and indwelling urinary catheters, necessitating targeted preventive measures. The innovations of this study are as follows: (1) Through dynamic monitoring, we systematically evaluated the distribution and antibiotic resistance trends of pathogens, providing a crucial foundation for understanding the evolutionary patterns of antimicrobial resistance; (2) By focusing on the annual variations of different types of pathogens, we established a relatively comprehensive antimicrobial resistance surveillance system; (3) The analysis of risk factors offers significant guidance for the formulation of future clinical prevention strategies. This study has several limitations, including its single-center design and relatively limited sample size, which may affect the representativeness and generalizability of the results. Moreover, as this study is a retrospective analysis, potential biases may arise from the acquisition of certain clinical data. Future research will involve a multicenter collaborative study with an expanded sample size and the implementation of a prospective cohort study to minimize bias.

## Data Availability

The original contributions presented in the study are included in the article/Supplementary Material, further inquiries can be directed to the corresponding author.
